# Typical imaging finding of hepatic infections: a pictorial essay

**DOI:** 10.1007/s00261-020-02642-z

**Published:** 2020-07-26

**Authors:** Sonaz Malekzadeh, Lucien Widmer, Faezeh Salahshour, Bernhard Egger, Maxime Ronot, Harriet C. Thoeny

**Affiliations:** 1 Department of Diagnostic and Interventional Radiology, Fribourg Cantonal Hospital, Fribourg, Switzerland; 2 Faculty of Medicine, University of Fribourg, Fribourg, Switzerland; 3 Department of Diagnostic and Interventional Radiology, Imam Khomeini Hospital, Tehran, Iran; 4 Department of Surgery, Fribourg Cantonal Hospital, Fribourg, Switzerland; 5 Department of Radiology, AP-HP, HUPNVS, Beaujon Hospital, Clichy, France

**Keywords:** Hepatic infection, Liver imaging, Liver infection

## Abstract

Hepatic infections are frequent in clinical practice. Although epidemiological, clinical and laboratory data may suggest hepatic infection in certain cases, imaging is nearly always necessary to confirm the diagnosis, assess disease extension and its complications, evaluate the response to treatment, and sometimes to make differential diagnoses such as malignancies. Ultrasound (US) is usually the first-line investigation, while computed tomography (CT) and magnetic resonance imaging (MRI) provide better characterization and a more precise assessment of local extension, especially biliary and vascular. The purpose of this article is to describe the typical features and main complications of common hepatic infections. Familiarity with the radiological features of this entity can help suggest the correct diagnosis and the need for further studies as well as determine appropriate and timely treatment.

## Introduction

Hepatic infections are relatively common. The prognosis varies and depends on the clinical context, the etiology of the underlying infection as well as when appropriate treatment is started. The clinical presentation ranges from chronic indolent forms to more aggressive lesions that are associated with a high mortality, especially in vulnerable or immunocompromised patients. The non-specific clinical symptoms of liver infections, including fever, abdominal discomfort, and nausea, highlight the importance of imaging with ultrasound (US), computed tomography (CT), and magnetic resonance imaging (MRI) to obtain a correct and prompt diagnosis. Although the imaging features of liver infections may be characteristic and sometimes lead directly to a correct diagnosis, clinical, laboratory and imaging findings are usually needed to make a final diagnosis. Imaging-guided fine-needle aspiration may also occasionally be required. Besides its central diagnostic role, imaging is important during follow-up of hepatic infections to monitor response to treatment.

The aim of this pictorial review article is to describe the most common imaging features of hepatic infections, categorized into bacterial, fungal, viral, and parasitic infections (Table 1). We also describe typical radiological findings to differentiate infections from other pathologies. Typical imaging features are summarized in Table 2. Chronic viral liver infection is beyond the scope of this article and will not be discussed.

**Table 1:** Overview of infectious agent, sub-classifications, and organisms

Types of infectious cause	Sub-classifications	Organisms
Bacterial	Pyogenic abscess	Polymicrobial *Escherichia coli* *Klebsiella pneumoniae*
	Bacterial granulomatous disease	*Mycobacterium tuberculosis* *Brucella* species *Bartonella* species
Acute viral		Hepatitis A, B, C, D and E virus Human immunodeficiency viruses
Fungal		*Candida albicans* *Histoplasma capsulatum*
Parasitic	Infection of the hepatic parenchyma	*Echinococcus* species *Entamoeba histolytica*
	Infection of hepatic vessels	*Schistosoma* species
	Infection of bile ducts	*Fasciola* species *Ascaris lumbricoides* *Clonorchis sinensis*

**Table 2:** Typical imaging findings of main acute hepatic infections

Hepatic infection	Typical imaging findings	
Pyogenic	Uni/multilocular lesion, multiple tiny lesions coalesce to form a larger lesion (cluster sign), “double target” sign	
Tuberculosis	Hepatomegaly, multiple diffuse tiny lesions (miliary form), a non-specific unilocular lesion	
Brucellosis	Hepatomegaly, multiple diffuse tiny lesions with possible thick peripheral enhancement, unilocular lesion with central calcification	
Bartonellosis	Multiple non-specific lesions up to 3 cm	
Hepatitis viruses	Hepatomegaly, “starry sky” sign on US, periportal edema, hepatic hilum lymph node enlargement, gallbladder wall thickening	
HIV	Hepatomegaly, periportal lymph node enlargement, HIV cholangiopathy presented with papillary stenosis, long extrahepatic bile duct stenosis and irregular aspect of intrahepatic bile ducts	
Candidiasis	“Wheel-within-a-wheel” sign, “bull’s eye” sign, focal fibrosis on area of prior inflammation, focal scars and calcifications	
Histoplasmosis	Multiple non-specific small nodules	
Cystic echinococcosis	CE	Unilocular anechoic well-defined lesion with imperceptible wall
	CL1	Snowstorm sign
	CL2	Rosette sign
	CL3a	Water-lily sign
	CL3b	Cystic lesion containing multiple daughter cysts
	CL4	Ball of wool sign
	CL5	Partially or entirely calcified cyst
Alveolar echinococcosis	Type 1	Multilocular small cysts without solid component
	Type 2	Multilocular small cysts with solid component
	Type 3	Solid component which envelop large cyst and multiple small cysts
	Type 4	Solid component without cystic part
	Type 5	A unilocular large cyst
Amebic	“Double target” sign, lesions with central hypoattenuation slightly more attenuating than water on CT	
Schistosomiasis	Hepatomegaly, periportal fibrosis, “turtle back” or “tortoise shell” sign	
Fascioliasis	Patchy and linear ill-defined subcapsular lesions converging from the hepatic capsule toward the hepatic hilum, focal thickening and enhancement of Glisson capsule, thickening and dilatation of biliary ducts	
Ascariasis	Tubular structures in the biliary tree corresponding to adult worms, hepatic abscess as non-specific focal lesion	
Clonorchiasis	Mild diffuse peripheral intrahepatic bile duct dilatation reaching the subcapsular area with relative sparing of extrahepatic bile duct, thickening of bile duct wall with increased periductal enhancement, stenosis of intrahepatic bile ducts	

*HIV* Human immunodeficiency virus, *EBV* Epstein–Barr virus, *US* ultrasound, *CT* Computed tomography

## Bacterial infections

### Pyogenic liver abscess

Although pyogenic abscesses are often polymicrobial, *Escherichia coli* and *Klebsiellae pneumoniae* are the most frequently isolated pathogens [1, 2]. While the cause of pyogenic abscess can usually be determined, no obvious cause is found in up to 20% of cases, which are known as cryptogenic [3, 4]. The most common cause of a cryptogenic pyogenic abscess is the hypervirulent *K. pneumoniae,* which is associated with aggressive inflammatory disease and additional sites of infection in other organs [5, 6]. Surprisingly, it has a more favorable outcome than pyogenic abscesses, mainly because the former occur in immunocompetent patients [7]. Four main mechanisms can favor hepatic abscesses. First, they can be the result of hematogenous dissemination of gastrointestinal infections via the portal vein or disseminated sepsis via the hepatic artery. Bile infection, favored by duct obstruction from various etiologies including stones, neoplasms, and strictures (ascending cholangitis, pancreatic cancer, inflammatory bile duct diseases) is frequently observed. Moreover, biliary stents and bilio-digestive anastomosis are also iatrogenic predisposing factors for pyogenic liver abscesses [8, 9]. Finally, hepatic infection by continuity, such as hepatic abscess from cholecystitis or direct introduction of bacteria into the liver parenchyma, such as during hepatic biopsy or surgery, and superinfection of pre-existing hepatic lesions, e.g., cysts or necrotic liver lesions, are other routes of liver abscesses [10]. Classically, pyogenic liver abscesses are pus-containing uni- or multilocular lesions surrounded by a fibrotic capsule.

#### Ultrasonography

On US, the appearance varies depending on the size and content of the abscess and ranges from a well or ill-defined tiny hypoechoic nodule to a large hypoechoic lesion with septa and debris [11].

#### Computed tomography

**Fig. 1:**
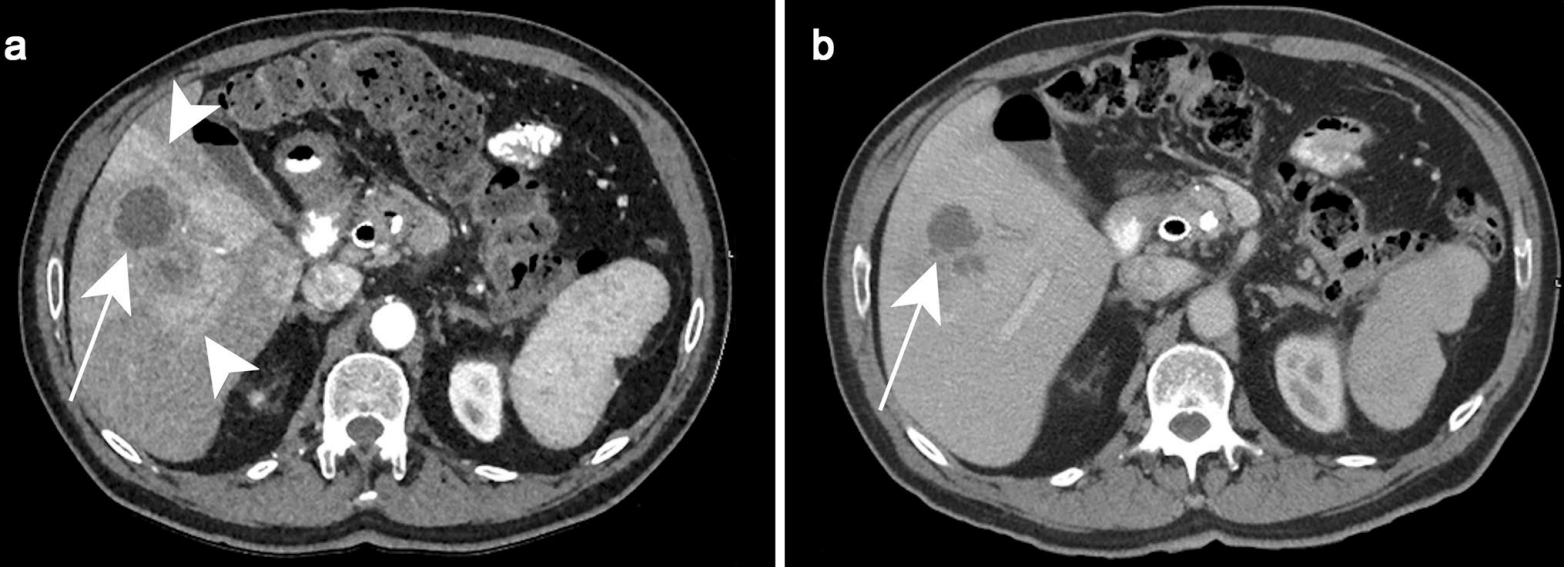
Pyogenic abscess in a 60-year-old female patient with a history of chronic pancreatitis who presented with asthenia and fever. Axial arterial phase (**a**) and portal venous phase (**b**) contrast-enhanced CT show small clustering lesions with a dominant hypoattenuating lesion in segment V of the liver (white arrows) corresponding to pyogenic hepatic abscesses. Altered perfusion disorder is observed as geographic areas of hyperattenuation peripheral to the hepatic abscesses (white arrowheads), clearly visualized on arterial phase (**a**)

The same appearance may be observed on CT with the characteristic “double target sign,” defined as early arterial enhancement of the inner wall of the abscess and progressive enhancement of the outer layer [12]. The entire lesion is surrounded by segmental geographic or peripheral transient perfusion disorders, identified as regions with early arterial phase enhancement and iso-attenuation on portal venous and delayed phases [13]. These perfusion disorders are related to perilesional venule stenosis, due to edema and infiltration by inflammatory cells (Fig. 1). On CT pyogenic abscesses may also present as multiple tiny hypoattenuating lesions with peripheral rim enhancement that sometimes coalesce to form larger lesions, a feature referred to as the “cluster sign” [14] . This is a typical feature in abscesses of biliary origin.

#### Magnetic resonance imaging

On MRI, the central pus is hyperintense on T2-weighted images (T2WI) and hypointense on T1-weighted images (T1WI), with impeded diffusion due to pus accumulation and the increased viscosity of pus on Diffusion-weighted (DW MRI) imaging. The inner and outer layers of the wall appear hypo- and hyperintense on T2WI, respectively. Although pyogenic abscesses usually appear to be fluid collections, they may also have a more solid appearance, mimicking primary or secondary hepatic tumors. This is often found in association with *K. pneumoniae* [15].

#### Differential diagnosis

The main differential diagnosis of pyogenic abscesses includes primary or secondary hepatic tumors and amebic abscess. It is important to note that transient perilesional enhancement, which is more frequently associated with a pyogenic abscess, helps exclude hepatic tumors. Pyogenic liver abscesses may also be complicated by hepatic or portal vein thrombosis with a reported incidence of up to 42% [16, 17]. Necrotic hepatocellular carcinoma associated with venous invasion can mimic a hepatic abscess complicated by cruoric venous thrombosis. However, venous thrombosis with luminal expansion, arterial phase intraluminal enhancement, and impeded diffusion of the venous structure suggests tumoral rather than cruoric venous thrombosis. Furthermore, the associated colon involvement supports amebic infection. However, the percutaneous approach is usually warranted for the diagnosis and therapeutic purposes.

### Tuberculosis

Hepatic involvement in tuberculosis can occur from pulmonary or miliary tuberculosis or less frequently via portal vein from gastrointestinal lesions [18]. Hepatic tuberculosis can be local (tuberculous primary complex with caseous necrosis of the hepatic hilar lymph nodes) or miliary, a part of a generalized disease. The latter is the most common form of liver tuberculosis. [19]. Tuberculoma can also develop and correspond to the enlargement and confluence of miliary foci or nodular development of tuberculous foci.

#### Ultrasonography

**Fig. 2:**
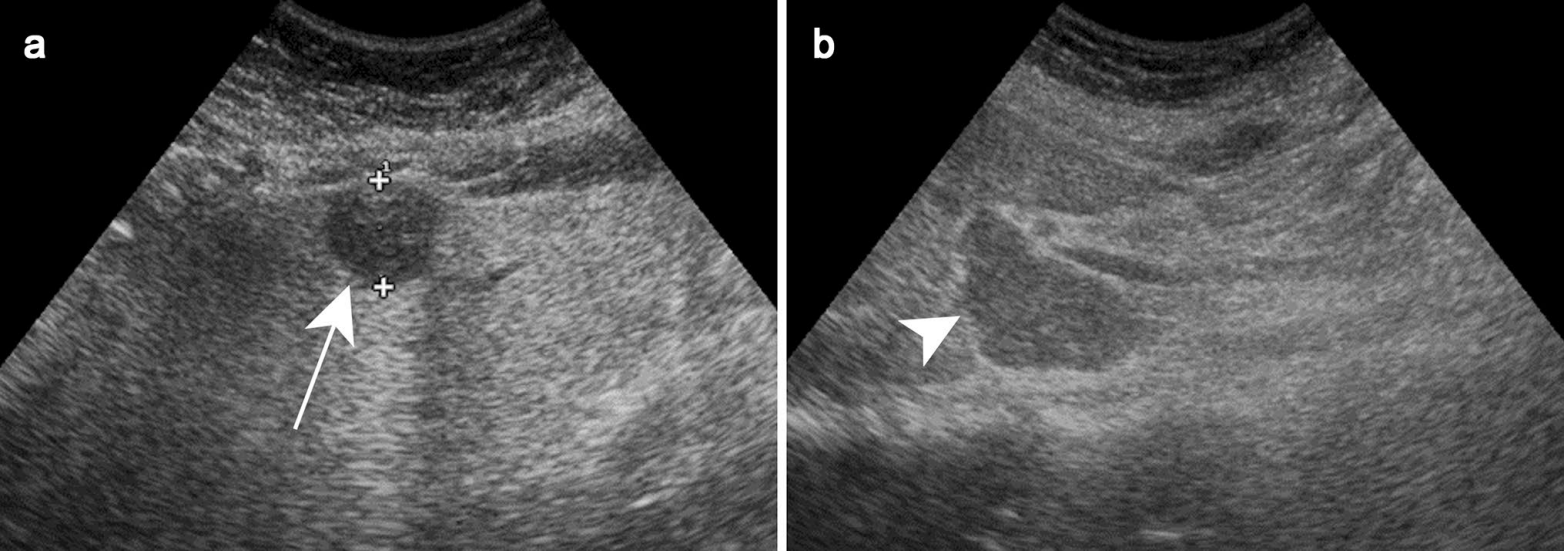
Liver tuberculosis in a 49-year-old female patient with asthenia and loss of weight without fever. Ultrasound **a** demonstrates a focal well-defined subcapsular hypoechoic lesion in segment III (white arrow). Enlarged lymph node (white arrowhead) is also observed in the porta hepatis (**b**) (Courtesy of Dr. Suzan Elhakiem, Ibn Sina Hospital, Khartoum, Sudan)

On US, the presentation of miliary hepatic involvement includes hepatomegaly with a diffuse hyperechoic aspect to the liver parenchyma with or without small diffuse hypoechoic lesions [20]. In the macronodular tuberculosis, single or multiple focal lesions with variety of appearance ranging from hyper- to hypoechoic lesions can be observed (Fig. 2a, b). Both hyper- and hypoechoic features are thought to represent different phases of disease corresponding to the degree of necrosis [21].

#### Computed tomography

On CT, the miliary form is observed as the multiple small hypoattenuating foci with discrete enhancing rim after contrast administration [22]. The macronodular lesions are detected as hypoattenuating lesions ranging from 14 to 45 HU on unenhanced CT, with tiny peripheral enhancement after contrast administration while the central part remains unchanged [23]. Calcifications can be observed in both miliary and macronodular forms [19].

#### Magnetic resonance imaging

On MRI, the miliary form is detected as multiple tiny lesion which are hypointense on T1WI and hyperintense on T2WI. The macronodular form presents hypo- or hyperintense central area on T2WI, with a hypointense rim [22, 24]. As observed with pyogenic abscess, liver tuberculosis can demonstrate impeded diffusion on DW MRI, making it difficult to differentiate from pyogenic abscess.

#### Differential diagnosis

The main differential diagnosis of miliary form includes lymphoma, metastatic lesions, sarcoidosis, and fungal infections. For the macronodular form, primary and metastatic hepatic lesions as well as pyogenic abscesses constitute the main differentials. Imaging is usually insufficient to make the definitive diagnosis and percutaneous biopsy is needed.

### Brucellosis

Hepatomegaly is a typical feature of hepatic abscess in brucellosis. A suppurative hepatic abscess is also a rare finding in these cases. Solitary abscesses normally present with a central calcification [25].

#### Ultrasonography

Hepatic abscesses from brucellosis may be solitary or miliary. Solitary lesions are seen as heterogeneous, well-delineated lesions, while miliary abscesses are seen as multiple hypoechoic hepatic subcentrimetric lesions which are difficult to be differentiated from tuberculosis, candidiasis or lymphoma.

#### Computed tomography

**Fig. 3:**
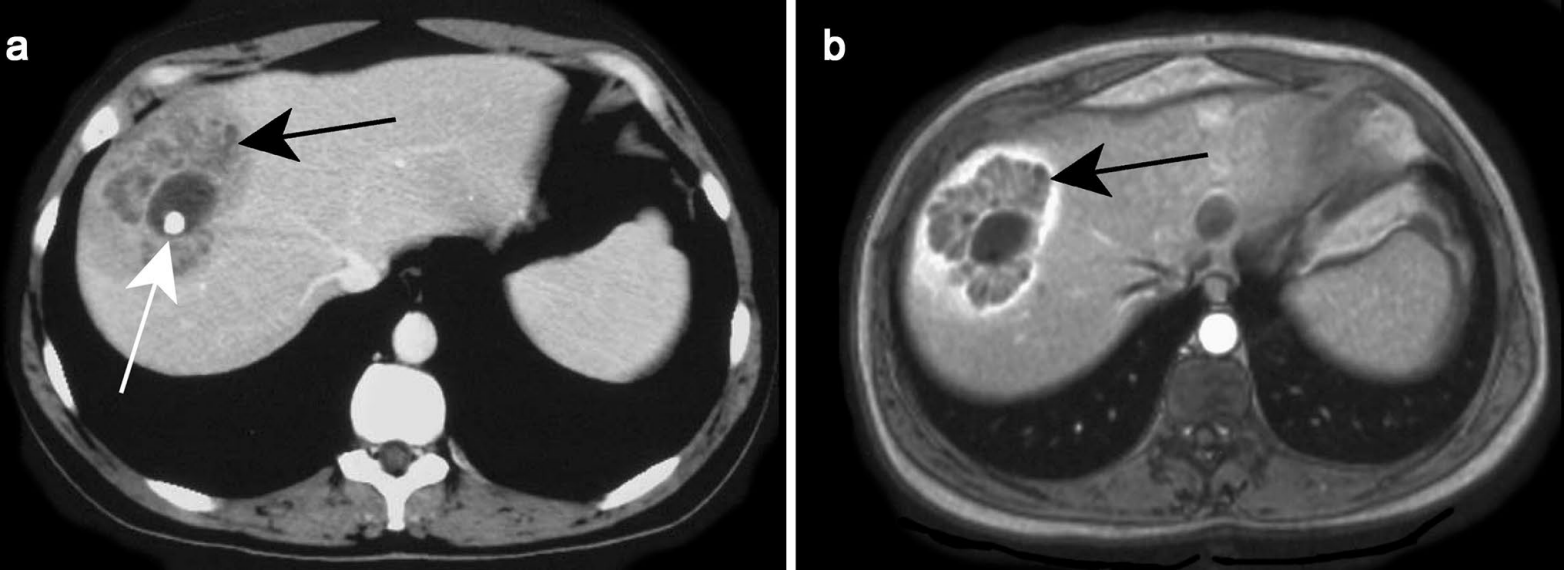
Brucellosis in a 42-year-old male patient with evening fever and sweating. Contrast-enhanced axial CT image **a** shows a heterogeneous lesion with enhanced contours (black arrow), showing a central hypoechoic fluid component and a calcium deposit (white arrow). Axial contrast-enhanced T1-weighted image **b** shows enhancement of the peripheral tissular areola (black arrow) and central saccular formation with fluid, surrounded by an intermediary heterogeneous component. Reprinted from Sisteron et al. [27], with permission from Elsevier

Hepatic abscesses are hypoattenuating with thick peripheral enhancement on contrast-enhanced CT (Fig. 3a). Perilesional transient perfusion disorder, like that found in pyogenic abscesses, may also occur with brucellosis.

#### Magnetic resonance imaging

These abscesses are hyperintense on T2WI on MRI. It is important to note that thickened peripheral enhancement, up to 15 mm, has been described in these abscesses on after contrast administration (Fig. 3b) [26, 27].

#### Differential diagnosis

When miliary, they should be differentiated from tuberculosis, candidiasis and lymphoma while pyogenic abscesses remain the differential diagnosis for solitary form.

### Bartonellosis

Bartonellosis, also known as “cat-scratch disease”, is usually associated with painful lymphadenopathy near the cat bite or scratch site. In the presence of liver involvement, multiple necrotizing granulomas, measuring up to 3 cm, can be detected throughout the liver parenchyma.

#### Ultrasonography

Necrotizing granulomas are seen as non-specific hypoechoic nodules throughout the liver parenchyma.

#### Computed tomography

**Fig. 4:**
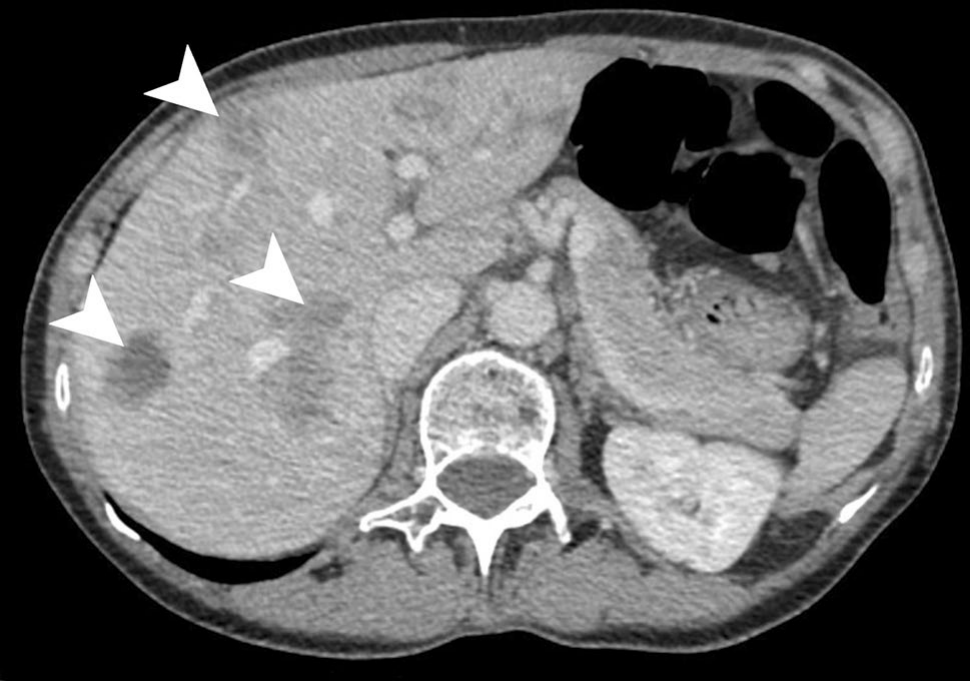
Bartonellosis in a 65-year-old female patient treated for autoimmune hepatitis. Axial contrast-enhanced CT demonstrates hypoattenuating ill-defined lesions are scattered throughout the liver parenchyma (white arrowheads)

These lesions are hypoattenuating on precontrast CT. These nodules may remain hypoattenuating after contrast administration (Fig. 4) or demonstrate iso-attenuation and sometimes rim enhancement [28].

#### Magnetic resonance imaging

On MRI, they are hypointense on T1WI and hyperintense on T2WI with the same enhancement as that of CT [29].

#### Differential diagnosis

Although it may be difficult to differentiate bartonellosis from lymphoma, fungal infection, sarcoidosis, tuberculosis or brucellosis on cross-sectional imaging, a history of cat contact in an immunocompetent child or young adult can be helpful.

## Acute viral infection

### Viral hepatitis

Acute hepatic viral infections are mostly caused by hepatitis A, B, C, D, and E viruses [30]. Ingestion of contaminated food or water and contact with blood or other body fluid of infectious person are the common ways of transmission. Although the radiological features of acute hepatitis are non-specific, imaging is usually performed to exclude other diseases with the same clinical signs, such as biliary obstruction or diffuse liver metastases.

#### Ultrasonography

On US, acute hepatitis usually presents with hepatomegaly, decreased hepatic echogenicity, as well as a relative increase in portal wall echogenicity, known as the “starry sky” sign [11].

#### Computed tomography

**Fig. 5:**
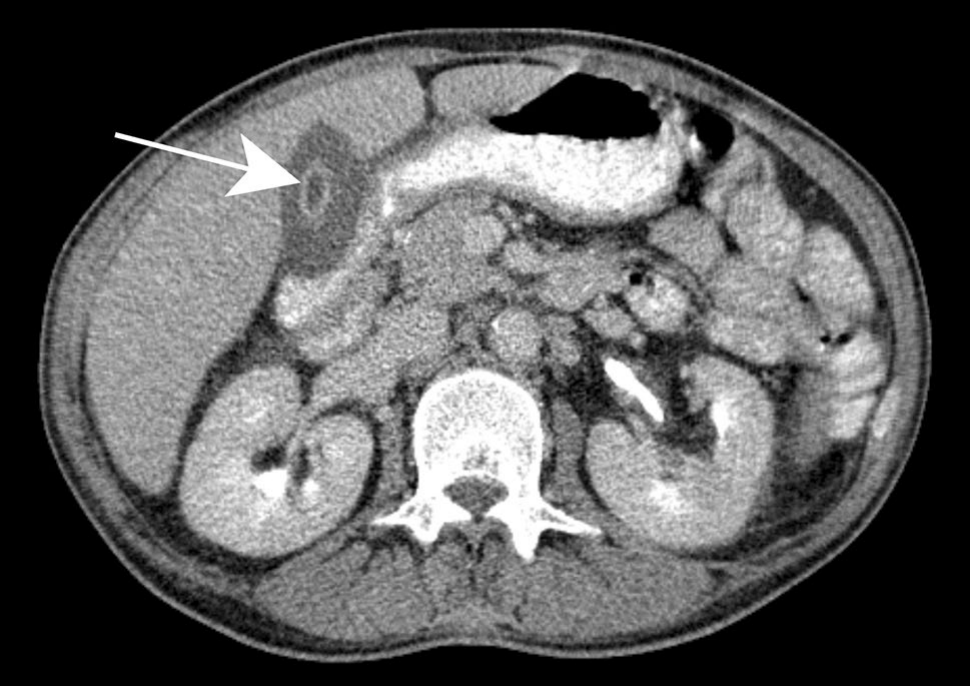
Acute viral hepatitis in a 59-year-old male patient with jaundice and elevated liver enzymes due to hepatitis A infection. Axial contrast-enhanced CT shows a contracted gallbladder with a thick hypoattenuating edematous wall and an enhancing mucosal layer (white arrow)

On CT, hepatomegaly, heterogeneous hepatic contrast enhancement on arterial phase images, well-defined parenchymal zones with low attenuation, periportal hypoattenuation, or hepatic hilum lymph node enlargement can be observed [31]. Gallbladder wall thickening may also be observed during acute hepatitis (Fig. 5) and should not be misinterpreted as acute cholecystitis [32]. Non-distended gallbladder and an absence of gallstones are the additional findings which suggest a diagnosis of acute hepatitis. Nevertheless, the liver may have a normal appearance on CT with serologically proven viral hepatitis.

#### Magnetic resonance imaging

The findings on MRI are the same as those on CT with a hyperintense periportal halo on T2WI and a hypointense T1WI image [33].

However, the diagnosis of viral hepatitis is mainly based on clinical and laboratory data rather than imaging findings.

#### Differential diagnosis

The imaging findings of viral hepatitis such as hepatomegaly and periportal edema are non-specific and differential diagnosis includes metabolic disease, passive hepatic congestion, autoimmune hepatitis and drug-induced hepatitis.

### Human Immunodeficiency Virus (HIV)

Liver involvement in patients with acquired immunodeficiency syndrome (AIDS) is not rare and frequently these patients suffer also from chronic viral infections such as hepatitis B and C.

#### Ultrasonography

On US, gallbladder wall thickening with biliary ducts wall thickening and dilatation can be encountered [11].

#### Computed tomography

Hepatomegaly and periportal lymphadenopathy are usually observed which are non-specific. In rare cases, focal steatosis and acalculous cholecystitis can also be seen [34].

#### Magnetic resonance imaging

**Fig. 6:**
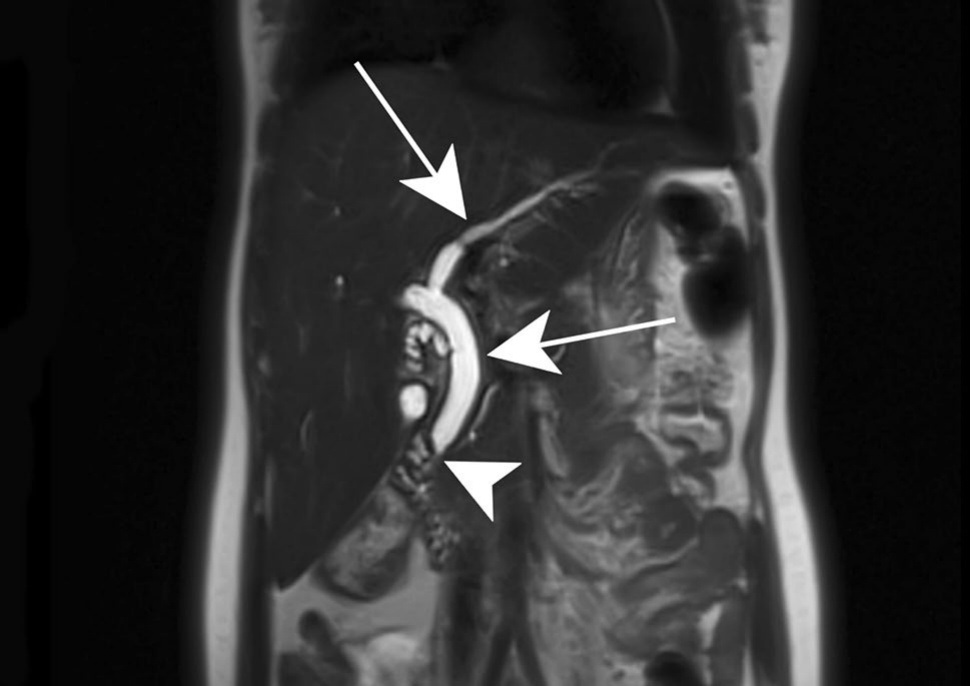
HIV-related cholangiopathy in a 16-year-old female patient. Coronal T2-weighted image demonstrates intrahepatic and extrahepatic bile duct dilatation (white arrows) due to papillary stenosis (white arrowhead), a common finding in this disease

Because HIV hepatopathies are frequently associated with biliary and pancreatic disorders, contrast-enhanced MRI with Magnetic resonance cholangiopancreaticography (MRCP) has been proposed in a single session to evaluate biliary tract lesions as well as liver and pancreatic parenchymal anomalies [35]. Imaging findings include biliary stenosis involving long extrahepatic segments, papillary stenosis (Fig. 6), and acalculous cholecystitis [35].

#### Differential diagnosis

As for viral hepatitis, the imaging findings for liver involvement in HIV such as hepatomegaly and periportal lymphadenopathy are non-specific. However, the primary sclerosing cholangitis is considered to be the main differential diagnosis for HIV cholangiopathy.

## Fungal infection

### Hepatic candidiasis

Invasive systemic candidiasis is a significant cause of morbidity and mortality in immunosuppressed patients, especially those receiving chemotherapy or with hematological malignancies.

#### Ultrasonography

Four US patterns of hepatosplenic candidiasis have been described [36]. The first pattern has a “wheel-within-a-wheel” appearance with a central hypoechoic area of necrosis and fungal debris, surrounded by a hyperechoic zone of inflammatory cells. A hypoechoic rim is found at the periphery, representing fibrosis. The second pattern is a bull’s eye configuration with a central hyperechoic nidus surrounded by a hypoechoic rim. In general, this pattern develops in patients with active fungal infection and a relatively normal white blood cell count. The third pattern is the most common and includes a uniformly hypoechoic nodule representing fibrosis that has developed in an area of prior inflammation, which is non-specific and can simulate metastases or lymphoma. The fourth pattern, which occurs in later stages of infection, consists of hyperechoic foci with different degrees of posterior acoustic shadowing, representing scars or calcifications.

#### Computed tomography

On CT, the microabscesses are seen as small, round, hypoattenuating lesions, in a miliary pattern [37]. Also, a “wheel-within-a-wheel” pattern, as observed by US, can be detected.

#### Magnetic resonance imaging

**Fig. 7:**
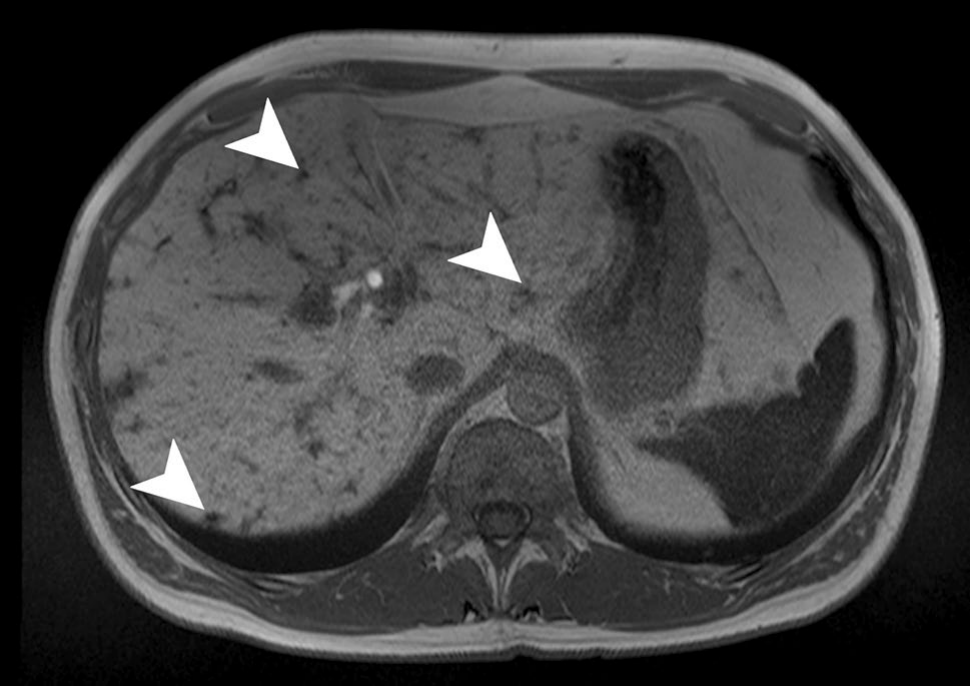
Candidiasis in a 65-year-old male patient with acute myeloblastic leukemia. Axial contrast-enhanced T1-weighted image on hepatobiliary phase illustrates multiple tiny hypointense lesions throughout the liver parenchyma (white arrowheads) (Courtesy of Dr. Luisa Paulatto, Beaujon Hospital, Clichy, France)

On MRI, the untreated nodules are markedly hyperintense on T2WI and minimally hypointense on T1WI (Fig. 7) with moderate enhancement after contrast administration [38]. After treatment, the microabscesses develop to granuloma with various imaging patterns according to the phase after treatment.

#### Differential diagnosis

Tuberculosis, sarcoidosis, metastases and lymphoma are the main diagnoses to be differentiated from hepatic candidiasis. A chest-X-ray may be conclusive to exclude tuberculosis and sarcoidosis. In patients with a known history of malignancy, the hepatic lesions can be likely metastases. However, a secondary fungal infection should also be considered in this group of patients. Lymphoma is usually associated with supra- and infra-diaphragmatic lymphadenopathies. Nonetheless, in some cases the percutaneous biopsy is conclusive for the diagnosis.

### Hepatic histoplasmosis

Histoplasmosis is caused by inhalation of *Histoplasma capsulatum* spores. It usually develops in immunodeficient patients, such as HIV-positive patients and transplant recipients [39]. The liver is rarely the primary site of infection but it is often involved in the course of a progressive disseminated disease, which usually originates in the lungs or upper respiratory tract. Imaging lacks sensitivity and specificity, and findings are similar to those in tuberculosis, candidiasis or other disseminated fungal diseases, with multiple small nodules in the liver parenchyma.

## Parasitic infections

### Infection of the hepatic parenchyma

#### Echinococcosis

*Echinococcus granulosus* and *Echinococcus multilocularis* cause cystic echinococcosis (CE) and alveolar echinococcosis (AE), respectively. While *E. granulosus* is more common, *E. multilocularis* is more invasive [40]. Infections occur by either ingestion of food or plants containing the eggs from the *Echinococcus* tapeworm or by direct contact with the main hosts, which are dogs (*E. granulosus*) and foxes (*E. multilocularis*) [41, 42]. The ingested embryos reach the portal venous system by invading the mucosal duodenal wall then embed the sinusoidal spaces and develop cysts.

#### *Echinococcus granulosus*

The mature cyst (i.e., hydatid cyst) of *E. granulosus* is composed of three layers. The outer layer or pericyst, mainly corresponds to the compressed adjacent hepatic parenchyma. The middle layer or ectocyst, is a translucent acellular layer allowing nutrition to pass to the endocyst, while the inner germinal layer produces the scolices, surrounding a fluid-filled central cavity [43, 44].

##### Ultrasonography

The appearance of CE on imaging, which is best evaluated by US, depends on the stage of cyst growth, classified by WHO into the six following subgroups:

- CL (cystic lesions): are well-defined, unilocular, anechoic lesions with an imperceptible wall.
- CE1: is an anechoic lesion with a perceptible double-layer wall that contains dependent low-level echos called hydatid sand. Hydatid sand (free scolices produced by the endocyst) is mobile when the patient changes position, which is referred to as the “snowstorm” sign [11, 42, 45].
- CE2: is a cystic lesion that contains multiple septa or multiple cystic lesions involving nearly the entire cystic cavity so that the walls of the cysts are very close to each other, with a “rosette” appearance.
- CE3a: in these cases the germinal layer is detached from the pericyst, which remains intact and is seen floating in the cystic cavity, known as a “water-lily” sign [41, 44].
- CE3b: is a cystic lesion that encases multiple daughter cysts. The daughter cysts are arranged peripherally in the cystic cavity which contains a solid-appearing matrix compared to the fluid in CE2.
- CE4: presents as a heterogeneous mass that ranges from hypoechoic to hyperechoic on US, with no identifiable daughter cyst.
- CE5: is a partially or entirely calcified cyst. When the cyst wall is calcified, it presents as a hyperechoic peripheral rim with acoustic shadow.

##### Computed tomography

**Fig. 8:**
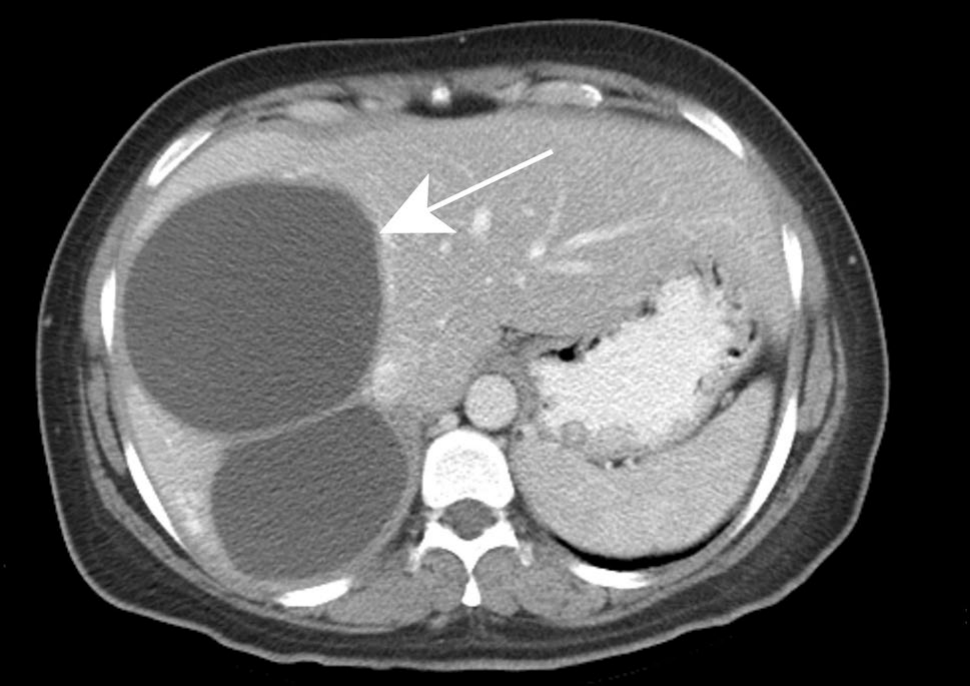
Cystic echinococcosis type CL in a 32-year-old female patient with upper abdominal discomfort. Axial contrast-enhanced CT image shows two large cystic lesions with thin walls in segment VII and VIII of the liver (white arrow). Ultrasound (not shown) revealed an anechoic cyst with a double layered wall, no internal daughter cyst or detached membrane

**Fig. 9:**
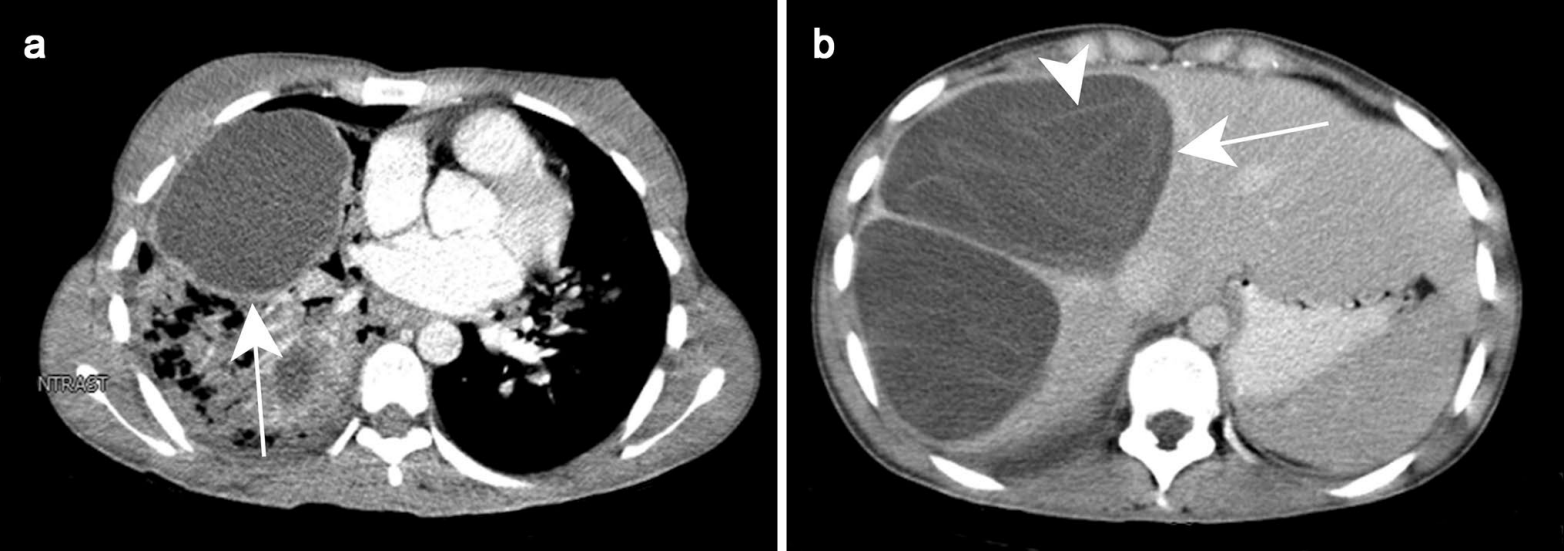
Cystic echinococcosis type CE3a in a 27-year-old female patient with cough. Axial contrast-enhanced CT of the lower pulmonary parenchyma **a** reveals a pulmonary cyst in the middle lobe (white arrow) associated with a partial consolidation of the right lower lobe. Axial contrast-enhanced CT of the upper abdomen **b** shows two hepatic cystic lesions (white arrow) with internal detached membranes (“water-lily” sign) (white arrowhead)

**Fig. 10:**
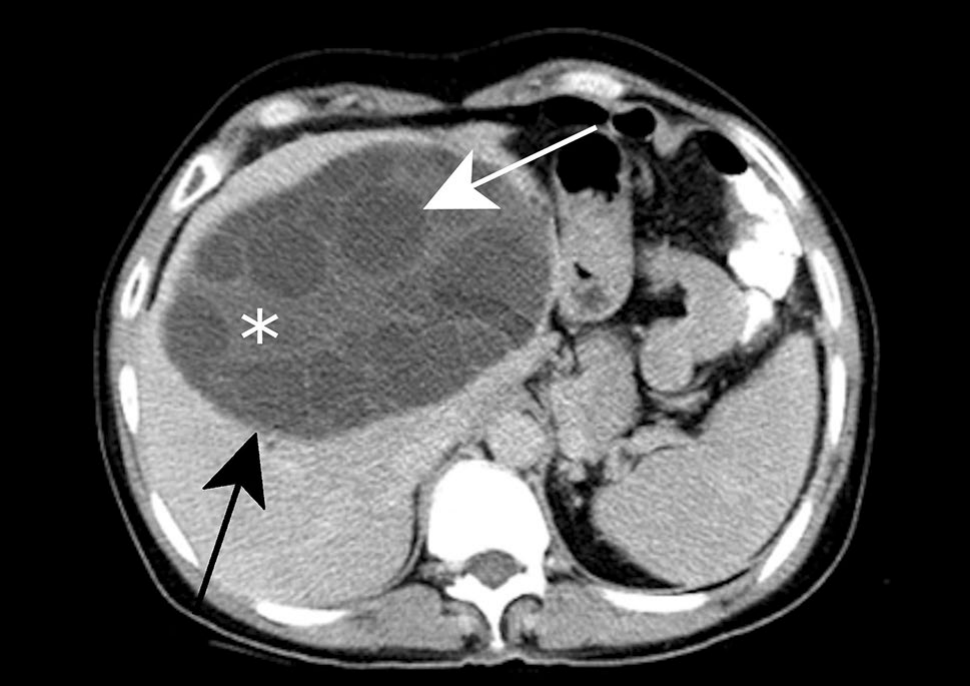
Cystic echinococcosis type CE3b in a 54-year-old male patient with right upper quadrant pain. Axial contrast-enhanced CT reveals a large hepatic cyst in right hepatic lobe (black arrow) with solid matrix in the center (asterisk) and peripherally located daughter cysts (white arrow)

**Fig. 11:**
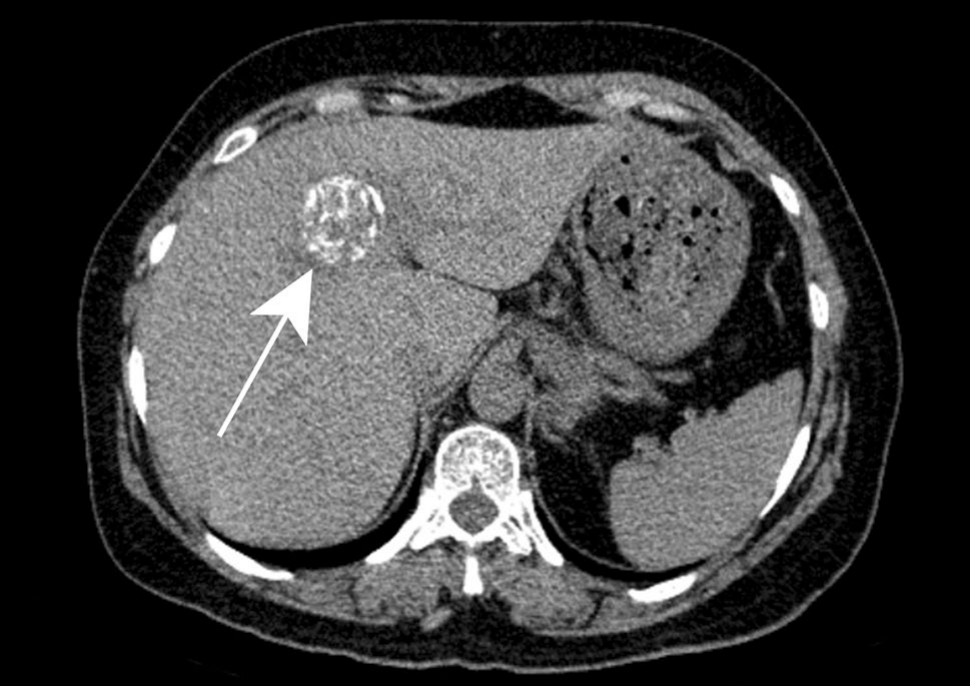
Cystic echinococcosis type CE5 in a 50-year-old female patient with an incidental solid mass reported on ultrasound. Axial precontrast CT shows a round highly calcified lesion in segment IV (white arrow)

On precontrast CT, the cyst wall usually appears as a hyperattenuating capsule that is nearly isoattenuating compared to the adjacent hepatic parenchyma following contrast administration [46]. CL is visualized as a well-defined, unilocular hypoattenuating lesion with thin wall (Fig. 8). The debris, when visible, shows no obvious contrast enhancement. The detached germinal layers are visible as serpiginous hyperattenuating structures (Fig. 9). The daughter cysts present as the hypoattenuating lesions with a density lower than the matrix of the cyst (Fig. 10). When the wall is calcified, it is well appreciated on CT (Fig. 11).

##### Magnetic resonance imaging

**Fig. 12:**
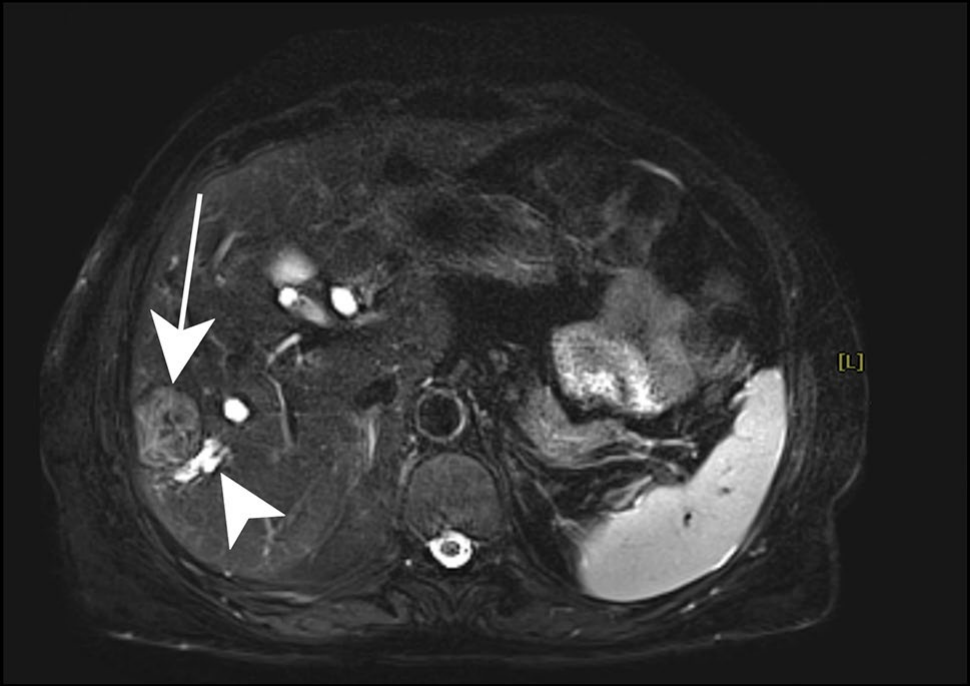
Cystic echinococcosis type CE4 in a 62-year-old female patient with upper abdominal pain. Axial fat-suppressed T2-weighted image shows a well-defined subcapsular moderately hyperintense lesion (white arrow) with a characteristic “ball of wool” sign. An adjacent dilated intrahepatic bile duct is also noted (white arrowhead)

On MRI the pericyst has a characteristic hypointense appearance, surrounding a markedly hyperintense T2WI and hypointense T1WI central cavity [44, 47, 48]. The daughter cysts are hyperintense and hypointense T2WI and T1WI, respectively, compared to the cyst matrix. The “ball of wool” sign, which is the characteristic feature of CE4, is a result of the detachment of the inner layer folding on itself so the lesion appears as a solid mass (Fig. 12). Calcifications are seen as the focal hypointense lesions on T2WI.

CL, CE1, and CE2 are active lesions, while CE4 and CE5 are inactive lesions. CE3 corresponds to transitional lesions which are degenerating cysts but containing viable protoscoleces [49, 50]. Hydatid cysts may be associated with complications including superinfection, communicating rupture, external rupture and the mass effect of large hydatid cysts. Superinfection is associated with a gas-fluid level or gas bubble in the hydatid cyst, frequently surrounded by areas of transient perfusion disorders in the surrounding hepatic parenchyma, such as in pyogenic abscesses [51]. Fistula with a hollow viscera or the tracheobronchial tree may also lead to gas-fluid levels which may be confounded with cyst superinfection [46, 52]. Communicating rupture is a cystic rupture into the biliary tree which may result in the passage of hydatid sand, a floating membrane from the germinal layer or daughter cysts into the biliary ducts, as well as fluid-fluid levels containing bile in the hydatid cyst. The latter are seen as fat droplets in the cyst with marked hypoattenuation on CT, and signal dropout on opposed phased gradient echo T1WI. This feature in not entirely specific for cystic rupture, since fatty transformation may occur in old cysts.

**Fig. 13:**
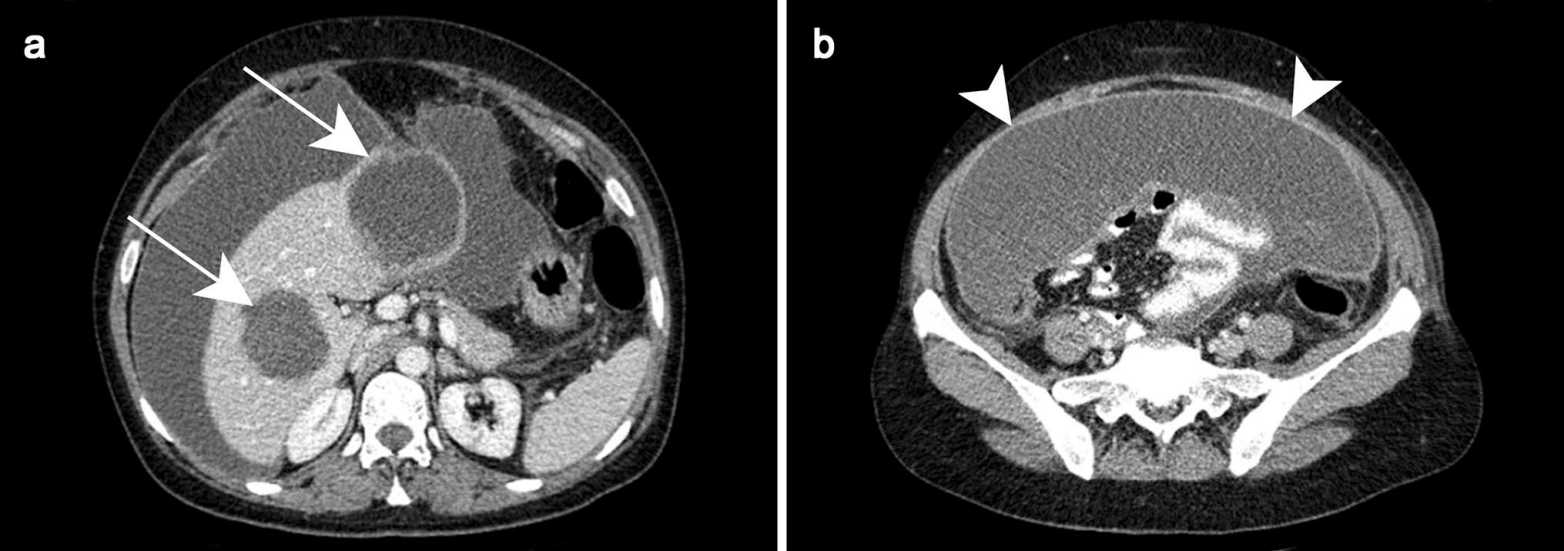
Peritonitis due to a ruptured hydatid cyst in a 45-year-old female patient with acute upper abdominal pain. Axial contrast-enhanced CT of the upper abdomen (a) demonstrates two hepatic cysts with irregular borders in the right and left liver lobe (white arrows); the latter reaches the anterior liver surface. Axial contrast-enhanced CT of lower abdomen (b) shows a significant amount of intraperitoneal fluid with a thickened enhancing peritoneum (white arrowheads)

**Fig. 14:**
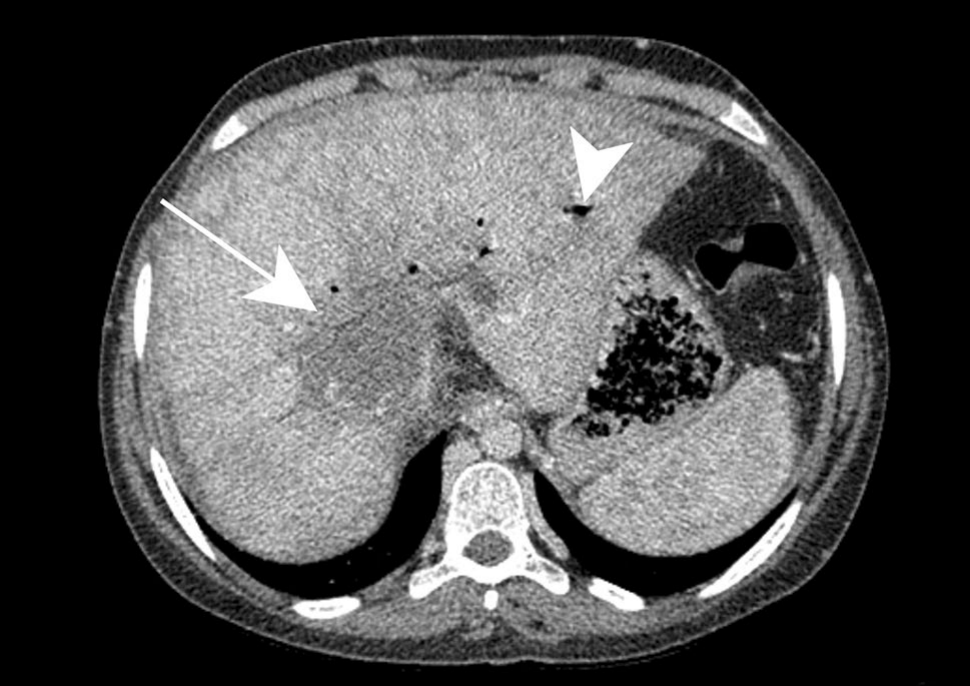
Budd-Chiari syndrome secondary to compression of liver out-flow by the hydatid cyst in a 20-year-old female patient. Hypertrophy of the caudate lobe with inhomogeneous mottled liver appearance is indicative of Budd-Chiari syndrome. Axial delayed phase contrast-enhanced CT shows a deformed cystic lesion in the deep portion of segments VII and VIII of the liver (white arrow) associated with pneumobilia secondary to previous sphincterotomy (white arrowhead)

External rupture is direct rupture of a cyst into the peritoneal or pleural cavity frequently via the bare area of the gastrohepatic ligament (Fig. 13). Finally, hydatid cysts can have a mass effect on the adjacent biliary or vascular structures. Chronic biliary obstruction and vascular compression, such as portal vein compression, can lead to hepatic segmental or lobar atrophy as well as secondary Budd-Chiari syndrome due to the mass effect on the hepatic veins (Fig. 14).

##### Differential diagnosis

Various congenital, inflammatory, infectious, and neoplastic cystic lesions can mimic different stages of CE. However, the typical imaging features of CE along with the serological information are usually helpful to discriminate CE from its counterparts.

The treatment of the CE depends on the stage of the cyst, including medical treatment, percutaneous approach recognized as PAIR (puncture, aspiration, injection, and reaspiration), surgical strategy, and watch-and-wait [53]. Medical treatment, PAIR, and catheterization are usually reserved for CE1 and CE3a, whereas modified catheterization and surgery are preferred methods for CE2 and CE3b. CE4 and CE5 can be controlled by watch-and-wait as they are considered to be inactive [50, 54].

#### Echinococcus multilocularis

AE includes small, multilocular confluent cysts associated with solid components that demonstrate exogenous growth invading the adjacent hepatic parenchyma. A large cystic component is also frequently observed. Small cysts include metacestodal vesicles, while large cysts are composed of liquefaction necrosis. Moreover, solid components encompass calcification and coagulation necrosis.

#### Ultrasonography

The two most frequent US findings of AE include a heterogeneous lesion with irregular borders and a large hypoechoic lesion. In the former, the heterogeneous lesion comprises the hypoechoic (necrosis and active parasitic tissue) and hyperechoic areas (fibrosis and calcification) with irregular borders indicating the invasive nature of the lesion while the latter is demonstrated as a central necrosis surrounded by hyperechoic fibrotic tissue [50].

#### Computed tomography

**Fig. 15:**
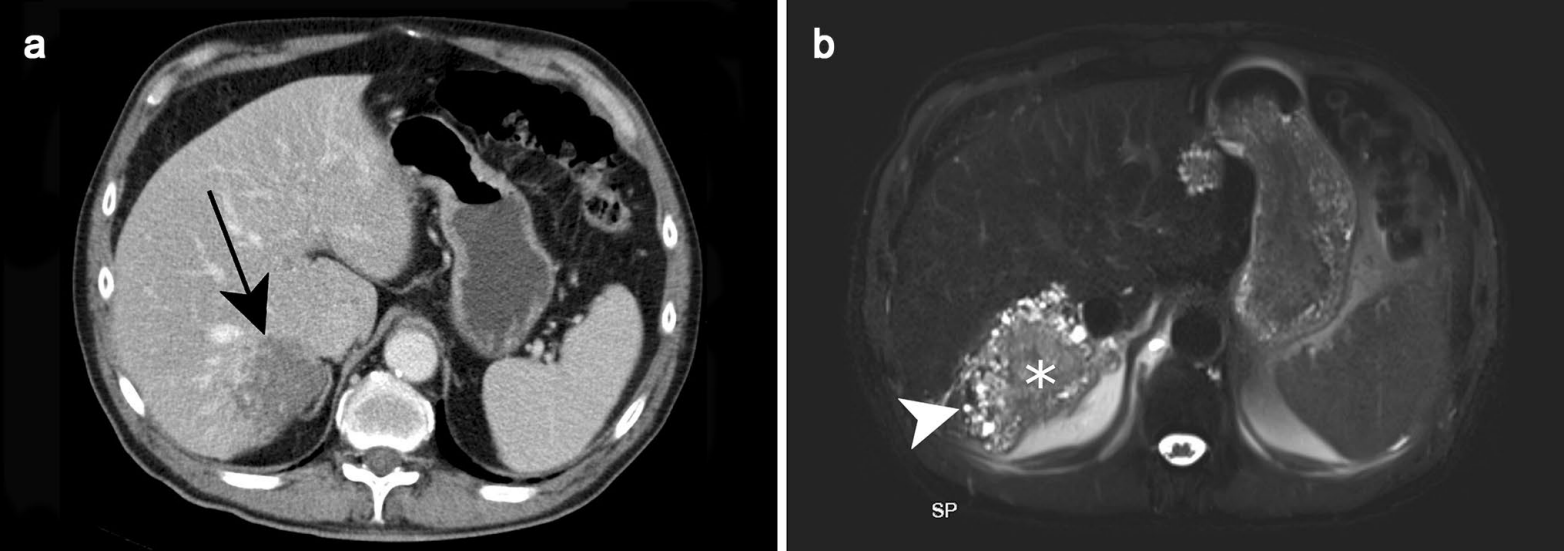
Alveolar echinococcosis incidentally detected in a 75-year-old male patient. Axial contrast-enhanced CT **a** shows an ill-defined subcapsular hypoattenuating lesion in segment VII of the liver (black arrow). Axial fat-suppressed T2-weighted image **b** further characterizes this lesion as multiple tiny cystic lesions (white arrowhead) surrounding a solid component corresponding to type 3 of alveolar echinococcosis

On CT, AE is usually presented as heterogeneous lesion containing hypoattenuating areas of necrosis and active parasitic tissue with scattered calcification with no obvious enhancement after contrast administration (Fig. 15a) [50].

#### Magnetic resonance imaging

Kodama et al. classified AE into five groups on MRI based on cystic and solid components, distribution and contrast enhancement (Table 2) [55]. Typical findings include peripheral arrangement of multilocular cysts and slight or no contrast enhancement of the solid component (Fig. 15b). While cystic components are markedly hyperintense on T2WI, the solid component can range from hypo- to hyperintense on T2WI [55, 56].

#### Differential diagnosis

The heterogeneous form of AE can be misinterpreted as primary and secondary hepatic malignancies and the large necrotic AEs should be differentiated from pyogenic and amebic abscesses.

### Amebic abscess

Liver amebic abscess is the most common site of extraintestinal involvement of amebiasis, the infection of the large bowel by *Entamoeba histolytica*. It occurs in less than 1% of patients with *E. histolytica* infection [11, 57]. Amebic abscess is usually a solitary unilocular cyst that is frequently located in the right hepatic lobe, especially the posterior segment.

#### Ultrasonography

**Fig. 16:**
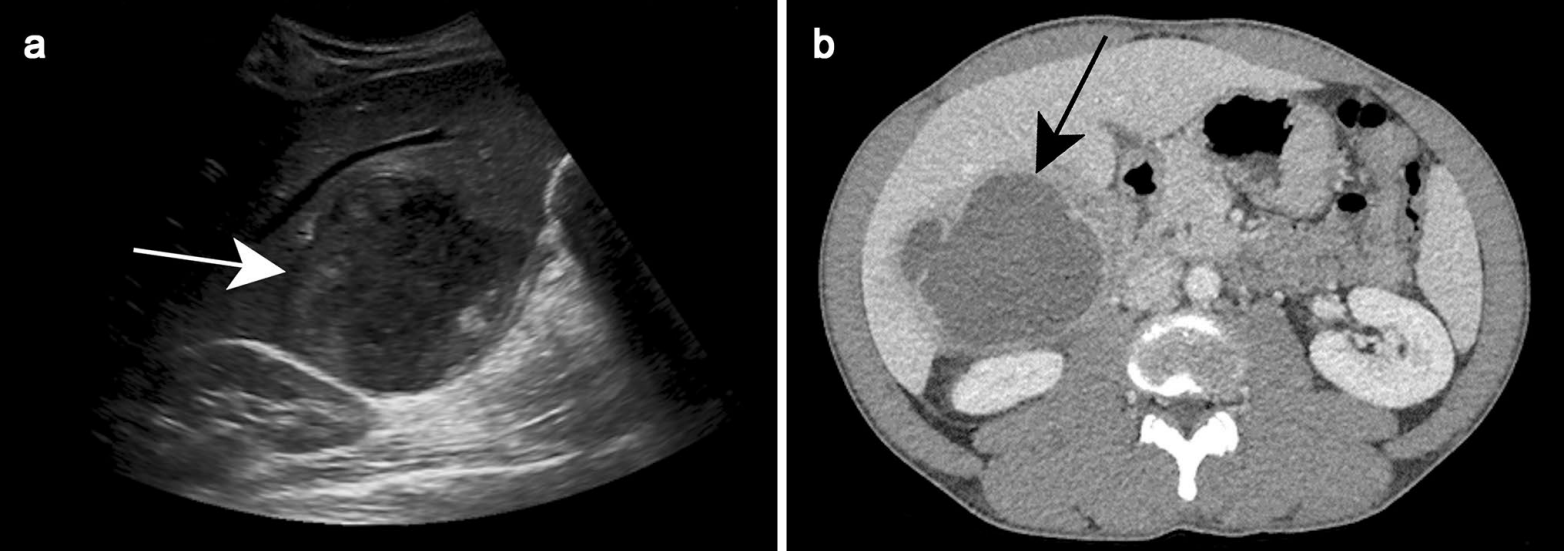
Amebic abscess in a 57-year-old male patient who presented with fever of unknown origin and right upper abdominal pain with a recent history of travel to Africa. Ultrasound **a** demonstrates a large relatively well-delineated lesion with a heterogeneous solid-appearing content (white arrow). Axial contrast-enhanced CT shows the “double target sign” (black arrow)

On US, it is demonstrated as a hypoechoic, well-delineated lesion containing low-level echoes that correspond to debris or hemorrhage (Fig. 16a).

#### Computed tomography

On precontrast CT, an amebic abscess is hypoattenuating but slightly more attenuating than water, and varies in density between 10 and 20 Hounsfield units [HU] with a thick peripheral capsule up to 1.5 mm in diameter [11, 58]. The capsule is enhanced after contrast administration and surrounded by peripheral hypoattenuation, known as the “double target sign,” similar to that observed with pyogenic abscesses (Fig. 16b) [43, 44].

#### Magnetic resonance imaging

On MRI, the central area is hypointense and hyperintense on T1WI and T2WI, respectively. The peripheral capsule is enhanced after contrast administration, and the lesion is surrounded by a hyperintense T2WI peripheral area [59].

#### Differential diagnosis

The appearance of an amebic abscess on imaging is nearly indistinguishable from that of a pyogenic abscess. However, a solitary abscess is more likely to be an amebic abscess compared to pyogenic abscess which is typically multiple. Also, the association of colon wall thickening that spares the ileum is highly suggestive of an amebic abscess. Furthermore, extrahepatic complications, such as pleural or pericardial effusion, and perihepatic collections, are more frequent with amebic than with pyogenic abscesses. Nonetheless, the definitive diagnosis is usually made through a combination of imaging, serological, microbial, and percutaneous aspiration data [60]. Metronidazole is the treatment of choice for amebic abscesses and aspiration or percutaneous drainage is considered for larger abscesses with high risk of rupture or in the case of failure to medical treatment [61].

### Infection of hepatic vessels

#### Schistosomiasis

Five species of S*chistosoma* cause human infection, and *S. mansoni* and *S. japonicum* are the most common causes of hepatic infection [62]. *Schistosomes* penetrate the skin to reach the bowel lumen where they lodge and release eggs into the mesenteric vein, gaining access to the portal system [44]. *Schistosoma* eggs cause a chronic inflammatory granulomatous reaction in the portal system, causing periportal fibrosis. Thus, the radiological features in the acute phase are non-specific, including hepatosplenomegaly and focal nodular liver lesions. In the chronic phase, fibrosis bands are observed surrounding the portal system. With *S. mansoni*, this is mainly observed when eggs are lodged in the proximal portion of the portal venous system while with *S. japonicum*, smaller eggs tend to lodge in the more distal portal veins [43].

#### Ultrasonography

**Fig. 17:**
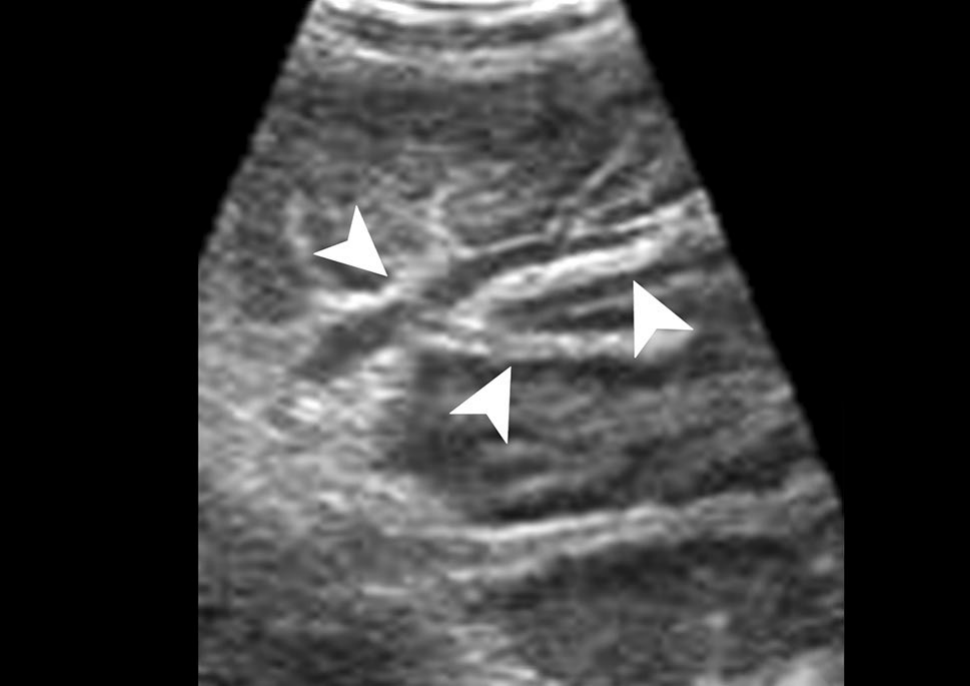
Hepatic schistosomiasis in a 20-year-old male patient with a history of gastrointestinal bleeding from 1 year ago. Ultrasound demonstrates a marked diffuse periportal thickening as a hyperechoic mantle encompassing the anechoic portal vein (white arrowheads) (Courtesy of Dr. Suzan Elhakiem, Ibn Sina Hospital, Khartoum, Sudan)

On US, the fibrosis bands are defined as a hyperechoic mantle encompassing the anechoic portal vein called the “bull’s eye” sign (Fig. 17) [11]. Common hallmarks of the chronic phase are a cirrhosis-like appearance with heterogeneous parenchyma and irregular contours.

#### Computed tomography

Periportal fibrosis presents as hypoattenuating bands on precontrast CT and with delayed phase contrast enhancement showing polygonal hypoattenuating structures surrounding areas of normal parenchyma [63]. The hypoattenuating peripheral septa observed with *S. japonicum* tend to calcify later in the disease and are seen as calcified septa, perpendicular to the hepatic capsule, called the “turtle back” sign or “tortoise shell” feature [63].

#### Magnetic resonance imaging

On MRI, periportal and polygonal fibrosis are hypointense on T1WI and hyperintense on T2WI with delayed contrast enhancement.

#### Differential diagnosis

A cirrhosis-like appearance of chronic schistosomiasis should be differentiated from other causes of cirrhosis. However, calcification and periportal fibrosis, which are typical findings in schistosomiasis, are not common with other causes of cirrhosis.

### Infection of bile ducts

#### Fascioliasis

*Fasciola hepatica* and *Fasciola gigantica* are parasites that are responsible for fascioliasis infection [64]. Sheep and cattle are the definitive hosts, while humans may be infected by ingesting contaminated water or freshwater plants [65]. There are two phases to fascioliasis infection, including a parenchymal (migratory phase) and biliary phase. During the parenchymal phase, juvenile flukes reach the peritoneal space by invading the small bowel wall, then reach the hepatic parenchyma by penetrating the hepatic capsule. They migrate to the biliary tree from the subcapsular space in linear tracts, converging toward the portal triads. During the biliary phase, the flukes mature in the small biliary ducts and produce eggs. Although the imaging findings depend on the phase of infection, both phases can be present simultaneously.

##### Ultrasonography

During the hepatic phase, US shows confluent hypoechoic ill-defined subcapsular lesions [66]. In the biliary phase, intra and extrahepatic bile duct dilatation is observed. A mobile intraductal parasite, when visible, is characteristic [67].

##### Computed tomography

**Fig. 18:**
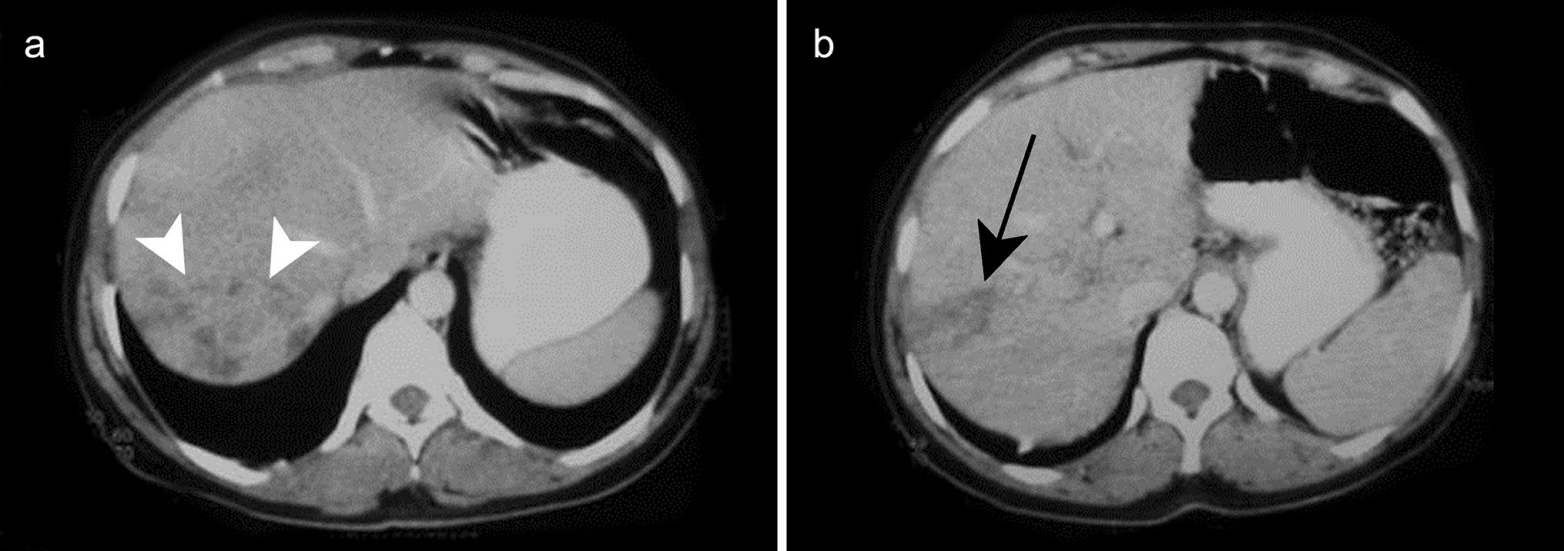
Fascioliasis in a 42-year-old female patient with right upper abdominal pain and low-grade fever. Axial contrast-enhanced CT (**a, b**) show patchy ill-defined hypoattenuating lesions with subcapsular (white arrowheads) and periportal distribution (black arrow)

On CT, ill-defined linear or patchy hypoattenuating subcapsular and periportal lesions that may converge from the hepatic capsule towards the hepatic hilum are observed (Fig. 18a, b) [68]. As observed on US, biliary ducts dilatation, gallbladder wall thickening and hilar lymphadenopathy can be seen. Focal thickening and hyperenhancement of the Glisson capsule may also be observed [69].

##### Magnetic resonance imaging

The lesions are hypointense on T1WI and hyperintense on T2WI, due to their inflammatory nature. Thickening and dilatation of the biliary tree similar to cholangitis can be observed during the biliary phase. The living, mobile parasite may sometimes be detected in the biliary tree as a biliary tree filling defect without contrast enhancement.

##### Differential diagnosis

Confluent tiny hypoattenuating lesions can mimic primary and secondary liver malignancy or pyogenic abscess. In addition, the biliary ducts wall thickening and enhancement observed with fascioliasis should be discriminated from other causes of cholangitis such as biliary stone.

#### Ascariasis

Ascariasis is a common infection caused by *Ascaris lumbricoides* in endemic areas. The adult worms mainly live in the jejunum, but may occasionally reach the ampulla of Vater due to altered small bowel motility [70]. Mechanical obstruction of the intrahepatic and common bile ducts by adult worms leads to cholangitis, cholecystitis, jaundice, and less frequently pancreatitis [71]. A hepatic abscess is also observed, although this is rare and thought to be due to a superinfection of the dead adult worm in the hepatic parenchyma [72].

##### Ultrasonography

On US, the radiological diagnosis of biliary involvement is mainly based on direct visualization of adult worms seen as a long tubular echogenic structure measuring up to 30 cm in the biliary tree. A longitudinal anechogenic line, representing the gastrointestinal tract of the worm in the center of the tubular structure, can also be seen [73]. A hepatic abscess presents as a non-specific hypoechoic focal lesion, usually with an ill-defined border [72].

##### Computed tomography

**Fig. 19:**
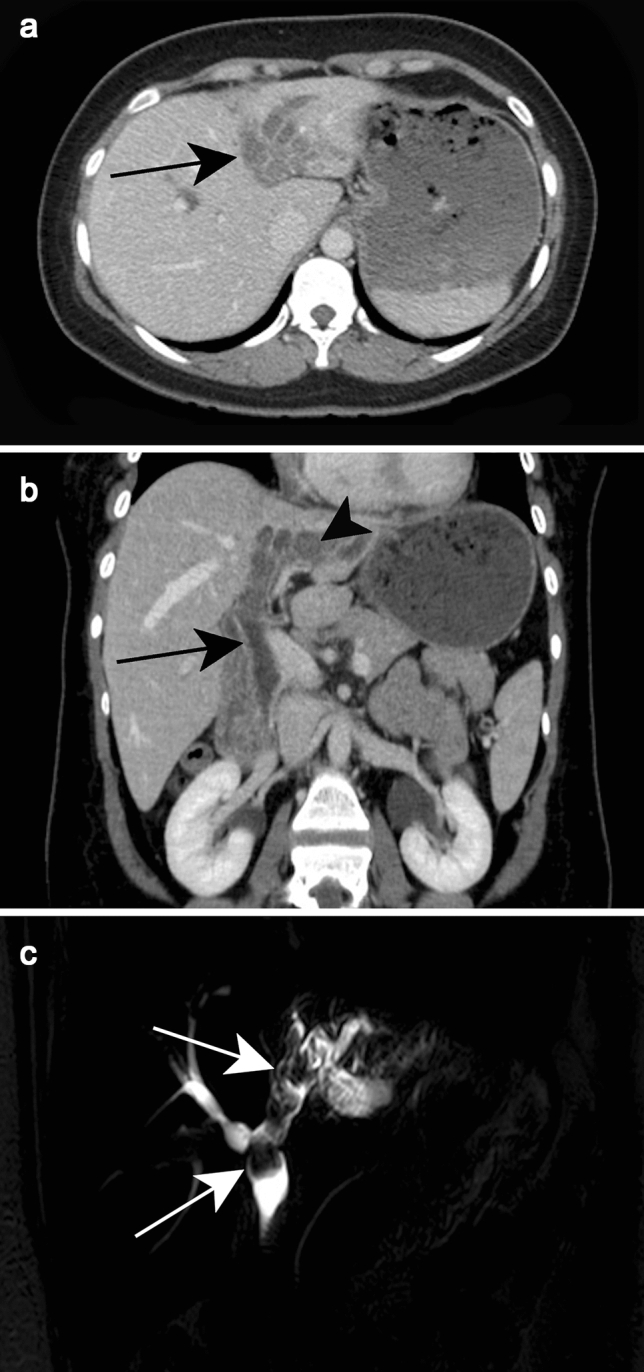
Ascariasis in a 36-year-old male patient. Axial and coronal reformatted contrast-enhanced CT (**a, b**) show intrahepatic and extrahepatic bile duct dilatation (black arrows). Note intrahepatic bile ducts filled with structures more attenuating than bile (black arrowhead), indicating adult worms. Oblique coronal single-shot fast spin-echo MR cholangiogram (**c**) shows adult worms as serpiginous and nodular filling defects in the left intrahepatic and extrahepatic bile ducts (white arrows)

Intra- and extrahepatic bile ducts dilatation can be depicted and the worm is seen as a linear filling defect in the bile duct (Fig. 19a, b)

##### Magnetic resonance imaging

As observed with other imaging modalities, the worm is seen as a linear filling defect in the bile duct on MRCP (Fig. 19c). Liver abscess resembles abscess with other pathogenic agents: a focal lesion hyperintense T2 and hypointense T1.

#### Clonorchiasis

Clonorchiasis is caused by chronic infection of *Clonorchis sinensis* following ingestion of raw freshwater fish [74]. When ingested, the cyst is freed by gastric juices and then reaches the biliary tree via the ampulla of Vater. Larva mature and lodge in the intrahepatic biliary ducts, although they may also reside in the extrahepatic bile ducts and gallbladder [74]. Flukes are leaflike structures ranging from 8 to 15 mm long and may lodge sporadically or grouped in the biliary tree, causing obstruction and an inflammatory reaction of the biliary epithelium. This chronic inflammatory reaction results in adenomatous hyperplasia, lymphocyte infiltration, ductal stenosis, and periductal fibrosis [75]. The imaging features of clonorchiasis are mainly based on the obstructive and inflammatory-induced effects.

##### Ultrasonography

Mild diffuse peripheral intrahepatic bile duct dilatation reaching the subcapsular area, with relative sparing of the extrahepatic bile ducts, is characteristic [76]. Hyperechoic bundles surrounding the intrahepatic bile ducts are present, indicating thickening of the wall ducts. Mature flukes may be observed as elliptical or filamentous hyperechoic structures in the biliary tree [77, 78]. Stenosis of the intrahepatic bile ducts can also be detected as the disease progresses.

##### Computed tomography

A thickened biliary duct with increased periductal enhancement is usually seen.

##### Magnetic resonance imaging

**Fig. 20:**
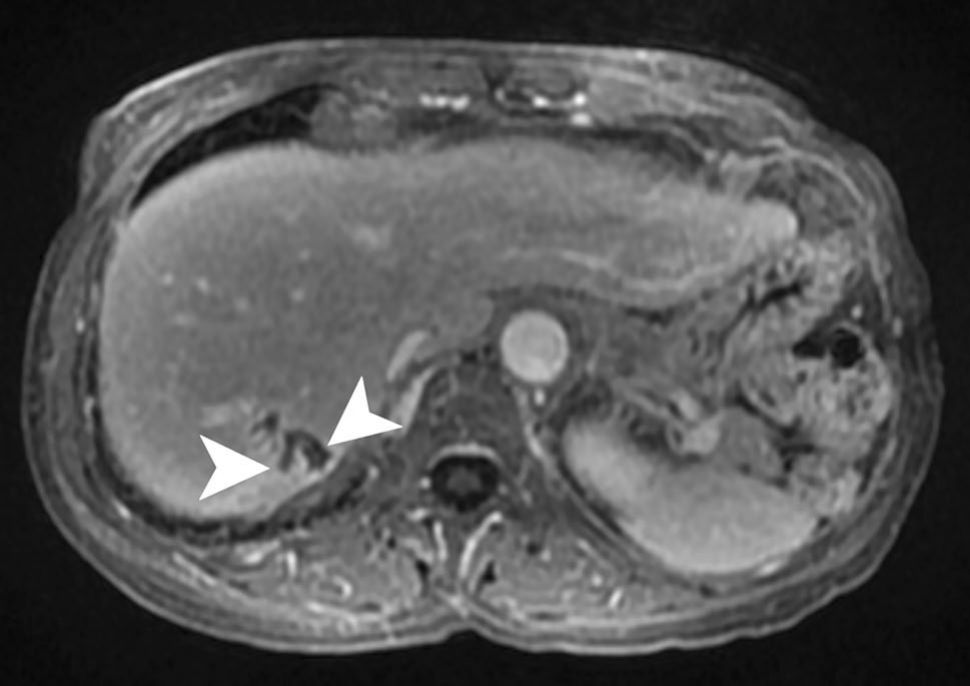
Clonorchiasis in a 74-year-old female patient with recurrent cholangitis. Axial contrast-enhanced fat-suppressed T1-weighted image shows intrahepatic bile ducts dilatation in segment VII reaching the subcapsular zone (white arrowheads)

As on US, intrahepatic bile ducts dilatation reaching the subcapsular area is observed (Fig. 20). MRCP shows elliptical or filamentous filling defects corresponding to mature flukes whose appearance can be differentiated from round or oval intraductal stones [76]. Cholangiocarcinoma is a well-known complication of clonorchiasis [78].

#### Differential diagnosis

It includes primary sclerosing cholangitis and recurrent pyogenic cholangitis.

## Conclusion

Imaging plays a central role in the diagnosis of hepatic infectious diseases. Although hepatic infections may have typical imaging features, additional epidemiological, clinical, and laboratory information is frequently needed to confirm the diagnosis. However, in some cases, imaging-guided aspiration is the only diagnostic tool that can determine the causative agent or eliminate non-infectious pathologies. Although different imaging modalities, including US, CT, and MRI, may identify certain unique features of hepatic infections, US is the primary diagnostic tool due to its low cost, the absence of radiation exposure and optimal biliary tree evaluation. However, in the presence of non-specific clinical symptoms, CT is usually performed to characterize hepatic lesions as well as to evaluate extrahepatic expansion or the presence of calcifications. MRI has also become increasingly popular due to superior contrast resolution. Furthermore, the entire biliary tree, in particular the peripheral intrahepatic bile ducts and the distal part of the common bile duct, may be visualized on MRCP, while such visualization is difficult to see on US and CT.
